# Correlation between US-PSV and 64-Row MDCTA with Advanced Vessel Analysis in the Quantification of 50–70% Carotid Artery Stenosis

**DOI:** 10.1155/2012/928638

**Published:** 2012-04-22

**Authors:** Matteo Stefanini, Eleonora Gaspari, Luca Boi, Costantino Del Giudice, Roberta Mastrangeli, Francesca Nucera, Giovanni Simonetti

**Affiliations:** Department of Imaging Diagnostic, Molecular Imaging, Interventional Radiology and Radiation Therapy, University Hospital “Tor Vergata,” 81 Oxford street, 00133 Rome, Italy

## Abstract

*Purpose*. To correlate ultrasonographic peak systolic velocity (US-PSV) and 64-row multidetector computed tomography angiography (MDCTA) with advanced vessel analysis (AVA) software in the quantification of 50–70% carotid artery stenosis. *Materials and methods*. 199 consecutive patients (247 arteries) with internal carotid artery (ICA) or third proximal bifurcation stenosis. Each patient was studied by duplex US (DUS) and 64-row MDCTA with AVA software. *Results*. DUS showed PSV measurements less than 125 cm/s in 51 carotid stenosis and a value greater than this in 196 arteries. 64-row MDCTA AVA software showed a grade of stenosis less than 50% in 42 carotid arteries while a greater 70% was found in 4 carotid arteries; then, carotid arteries with stenosis percentage between 50% and 70% were 201. Linear regression analysis showed a good linear correlation (*r* = 0.88) between MDCTA-AVA software percentage stenosis and PSV: between 50% grade of stenosis and PSV value corresponding to 133,6 cm/sec and between 70% stenosis and PSV value corresponding to 268 cm/sec. The sensitivity, specificity, positive predictive value(PPV), negative predictive value(NPV) of this analysis were 93%, 82%, 97%, 75%, respectively. *Conclusion*. Linear correlation between PSV data and grade of stenosis from 50% to 70% obtained with 64-row MDCTA AVA software. Main PSV value corresponding to 50% and 70% grade of stenosis at AVA analysis.

## 1. Introduction

Stroke is a dramatic medical problem: in fact when considered separatelyfrom other cardiovascular diseases, stroke is a third cause of death for females and fourth for male in the United States after heart disease and cancer [1]. It was shown that the number of stroke events in Europe would increase from 1.1 million per year in 2000 to more than 1.5 per year in 2025, based only on demographic changes [2].

Carotid artery atherosclerotic is an important etiological factor for ischemic stroke [3].Actually clinical decision making regarding intervention for carotid artery stenosis depends upon the grade (percentage) of stenosis. The large clinical trials on which many physicians rely while taking this decision, used digital substraction angiography (DSA) as a gold standard to evaluate the exact percentage of diameter stenosis [4–7]. The results of two large randomized trials-the North American Symptomatic Carotid Endarterectomy Trial (NASCET) and European Carotid Surgery Trial (ECST) have shown carotid artery endarterectomy to yield a considerable advantage in patients with 70–99% stenosis and a small benefit in symptomatic patients with 50–69% stenosis. Measuring according to the NASCET criteria is done by comparing the diameter of the lumen at the most stenotic part of the vessel to the diameter of the normal distal internal carotid artery (ICA) to the stenotic portion. The ECST method compares the lumen diameter of the most stenotic part to the estimated original diameter at the site of the carotid bulb [8–12]. In these studies, digital substraction angiography (DSA) was the gold standard for the evaluation of carotid stenosis but also associated with an increased risk of thromboembolic events and marked financial cost [13]. Consequently, numerous noninvasive imaging techniques are used in the evaluation of carotid artery degree of stenosis: magnetic resonance imaging (MRI), ultrasound sonography (US), and multidetector row computed tomography angiography (MDCTA) [14–22]. The aim of this study was to evaluate the correlation between US peak systolic velocity (US-PSV) and 64-row with MDCTA advanced vessel analysis (AVA) software in the quantification of 50–70% carotid artery stenosis in order to introduce the main PSV value to give a real indication for interventional treatment of symptomatic patients (with 70% stenosis) and asymptomatic ones (with 50% stenosis). 

## 2. Materials and Methods

### 2.1. Patients

From March 2007 to December 2010, 199 consecutive patients (247 arteries) with internal carotid artery (ICA) or third proximal bifurcation stenosis were retrospectively examined. In 24 patients, was bilateral stenosis observed. Each patient was studied by US-Duplex and 64-row MDCTA with advanced vessel analysis (AVA) software for two months.

169 patients were symptomatic and 31 asymptomatic. Neurological symptoms included transient ischemic attacks (*n* = 63), amaurosis fugax (*n* = 58), and minor stroke (*n* = 57). Mean patient age was 66 (range 52–81) years. The patients present several diseases as arterial hypertension, diabetes mellitus, dyslipidemia, and other cardiovascular risk factors (smoking, hypercholesterolemia, and obesity).

The study was approved by the ethics committee at our centre, and all patients gave written informed consent.

### 2.2. Carotid Duplex Ultrasound (DUS)

All carotid duplex scans (DUSs) were performed using equipment ATL HDI 5000 (Advanced Technology Laboratories, Bothel, Wash, USA) and Philips iU22 ultrasound system (Philips Healthcare, Best, the Netherlands) with a linear 4–7 MHz or 5–10 MHz duplex probes (Figures 2 and 4). All DUS carotid studies were performed by a radiologist with experience of more than 500 exams blind team CT results.

The carotid arteries were examined in supine position with the head slightly elevated and turned towards the contralateral side. Longitudinal and cross projections were performed on the entire extracranial section including common carotid artery (CCA), internal carotid artery (ICA), external carotid artery (ECA), and vertebral artery (VA) and using spectral analysis and B-mode ultrasound imaging (BMI) complemented with color flow mapping.

### 2.3. Pulsed Doppler Spectral Analysis

An initial Doppler sweep of the CCA, ICA, and the proximal ECA was performed to identify areas of increased velocity. While the Doppler beam angle was maintained under 60 degrees at all times, representative values of peak systolic velocity (PSV) were recorded (Figures 2(c) and 4(c)).

The ratio of PSV between ICA and CCA was calculated and recorded for each patient. The criteria used for diagnosing a significant stenosis and for grading of severity of carotid stenosis were based on previously published criteria from the University of Washington (Strandness criteria) reported in Table 1 [14, 15].

In each study, the highest PSV and ICA/CCA ratio were recorded.

### 2.4. Multidetector Row Computed Tomography Angiography (MDCTA) Examinations

All patients underwent multislice 64-row CT (GE, Medical systems, Milwaukee, WI, USA) (Figures 3 and 5) by a radiologist with large experience in CT assessment.

The CT scans covered the range between the aortic arch and the level of circle of Willis. A slice thickness of 1.2 mm (1 mm collimation, feed 5 mm/s) and a reconstruction interval of 1.0 mm were used.

The contrast agent (Ultravist 300 mgI/mL, Schering AG, Berlin, Germany) volume for CT angiography was 100 mL with a saline chaser bolus of 30 mL using a flow rate of 3,5 mL/s with a 1.3 mm (18 G) cannula through the antecubital vein. Smart prep technique positioning at the pulmonary artery was utilized to contrast agent injection. 

A separate workstation (ADW 4.0, GE, Medical systems, Milwaukee, WI, USA) was used for analysis of CTA images. An automated 3D CTA analysis based on AVA method was used (Figures 2(b), 4(b), and 4(c)) which provides an objective analysis of luminal cross-sectional area and of the smallest vessel diameter from a range between the level of maximal stenosis and from the level of reference distal to the carotid bulb.

**Figure 1:**
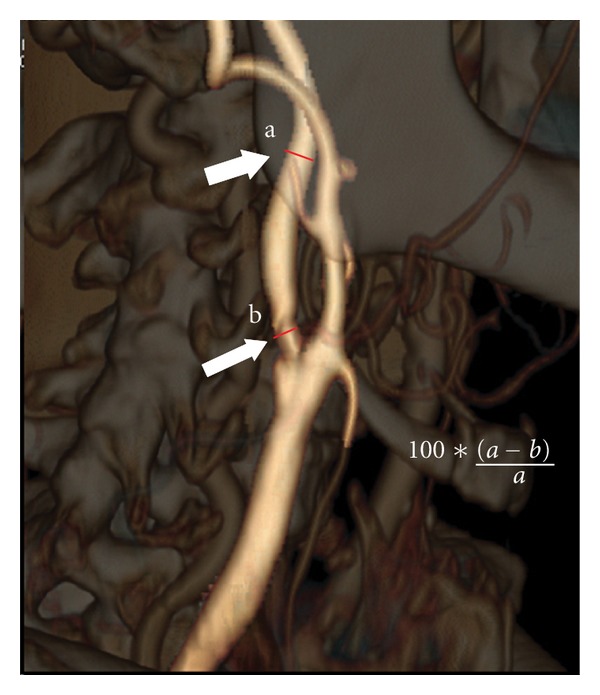
Volume rendering CT angiography showing measurement of internal carotid artery stenosis. Following the NASCET criteria, a measure of the ratio at the point of greatest stenosis (small white arrow) and at the normal part of the artery beyond the carotid bulb (big white arrow) was performed.

**Figure 2:**
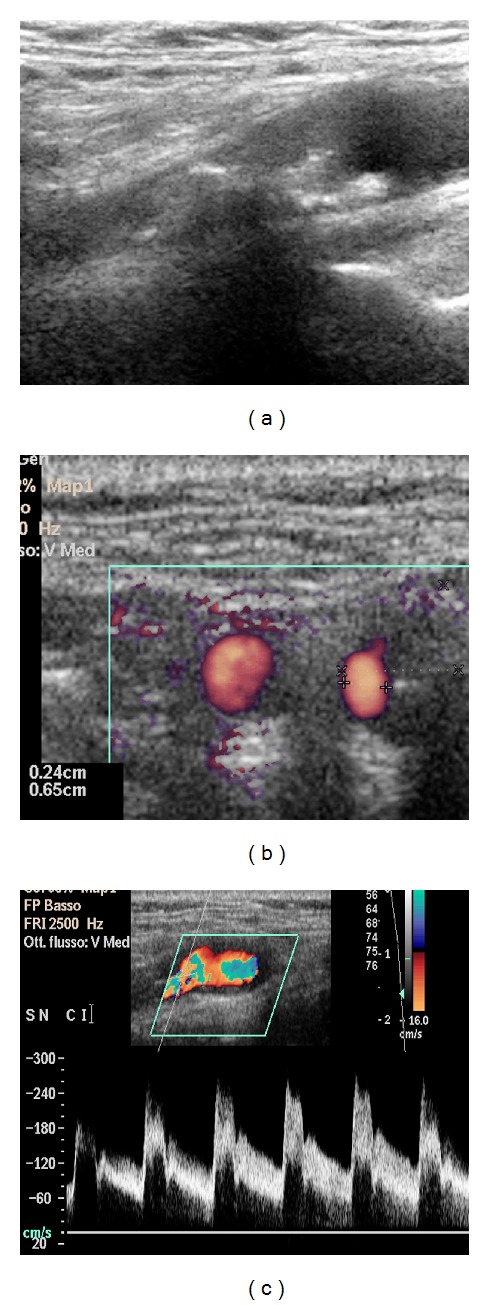
Left carotid stenosis demonstrated on Duplex US (Case  1) (a). Measurements of minimal lumen diameter (b) and Spectral US Evaluation with PSV Measure (c). The data obtained by Duplex US shows a high rate correlation with CTA measurements, with a stenosis of 80% evaluated according to strandness criteria and 79% with advanced vessel analysis (AVA) software at CT angiography.

**Figure 3:**
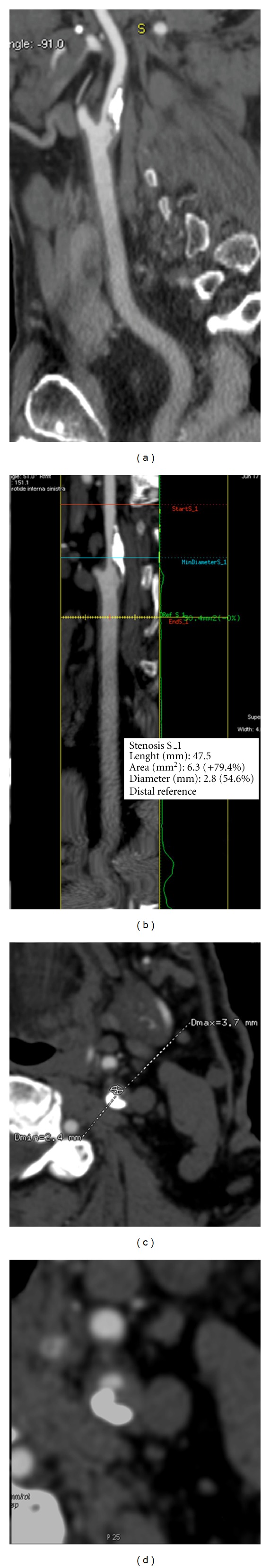
Left carotid stenosis demonstrated on CT angiography (CTA) (Case  1) with curved image (a) and lumen image produced by advanced vessel analysis (AVA) software (b). The curved image shows the plaque. The software analysis defines automatically the level of maximal stenosis. (c) regular cross-sectional image with the largest/smallest luminal diameter at the stenosis level. In this case the measurements on CTA show a minimum diameter of 2,4 mm with a 79% of stenosis degree. (d) Axial amage of left carotid artery.

**Figure 4:**
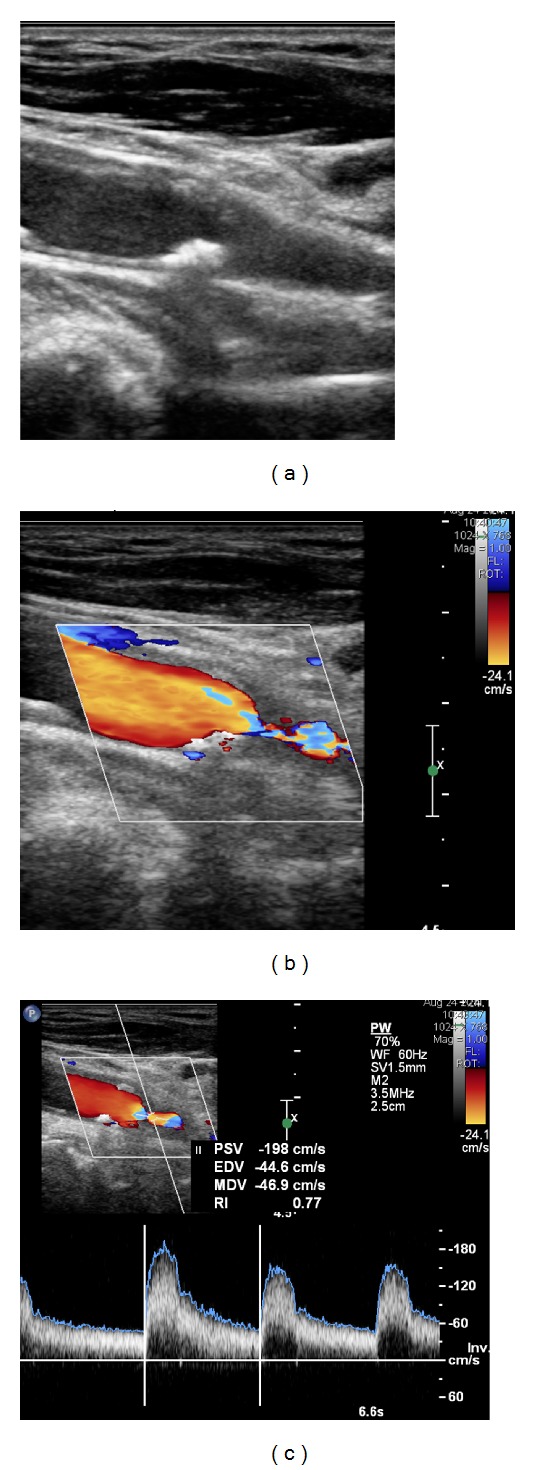
Right carotid stenosis demonstrated on Duplex US (Case  2) (a). Color image of the stenosis (b) and spectral US evaluation with PSV measure (c). According to the Strandness criteria, a stenosis of 50–79% was evaluated.

**Figure 5:**
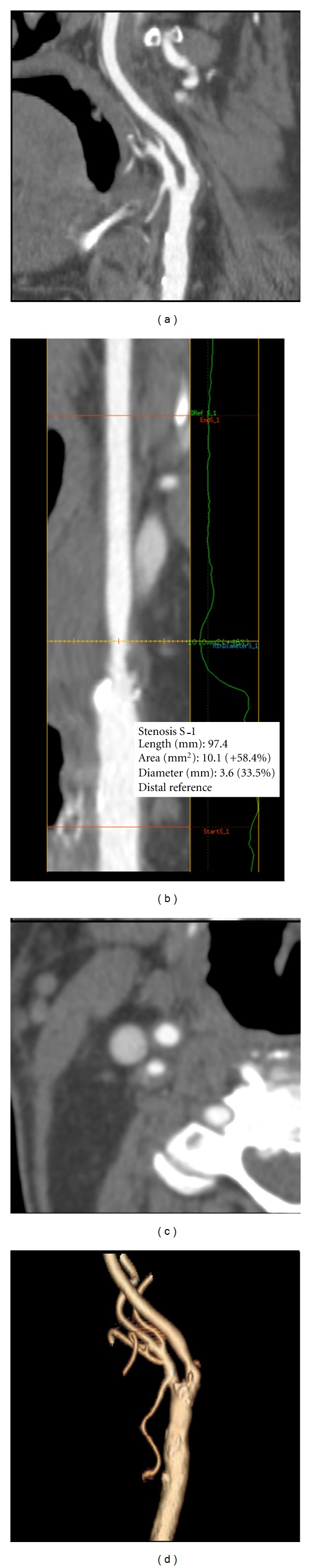
Right carotid stenosis demonstrated on CT angiography (CTA) (Case  2) with curved image (a) and lumen image produced by Advanced Vessel Analysis (AVA) software (b). The curved image shows the plaque. The software analysis defines automatically the level of maximal stenosis. Regular cross-sectional image showing the diameter at the stenosis level (c). In this case the measurements on CTA shows a minimum diameter of 3,6 mm with a 58.4% of stenosis degree. Volume rendering showing the right internal carotid artery (d).

The stenosis degree based on luminal area values was obtained by the following equation (Figure 1):

(1) $$100\times \;\frac{\text{area reference level}-\text{area maximal stenosis level}}{\text{area reference level}}\;.\;$$

### 2.5. Statistical Analysis

All duplex ultrasound velocity profiles, BMI and CTA diameter, and stenosis measurements were recorded into a computer database for analysis. Correlation of PSV at Duplex study and the grade of stenosis obtained by AVA analysis was evaluated using a linear correlation. From this correlation a mean value of PSV related, respectively, to 50% and 70% at AVA analysis was obtained. According to NASCET criteria stenosis >50% for symptomatic patients and >70% for asymptomatic patients were considered significant.

Sensitivity, specificity, positive predictive value (PPV) and negative predictive value (NPV) for the ability of Duplex scanning using Strandness criteria to correctly classify a significant stenosis were also evaluated considering CT results as gold standard.

## 3. Results

We studied 247 carotid arteries with Duplex ultrasonography (DUS), which showed PSV measurements less than 125 cm/s in 51 carotid stenosis and a value greater than this in 196 arteries. All patients were evaluated also with 64-row MDCTA AVA software measurements of linear percentage stenosis ranging from 40% to 80% (mean 60%). Of these stenosis was measured in 42 caratoid arteries by AVA software less than 50% while 4 carotid arteries had a grade of stenosis greater than 70%; then carotid arteries with stenosis percentage between 50% and 70% were 201.

**Figure 6:**
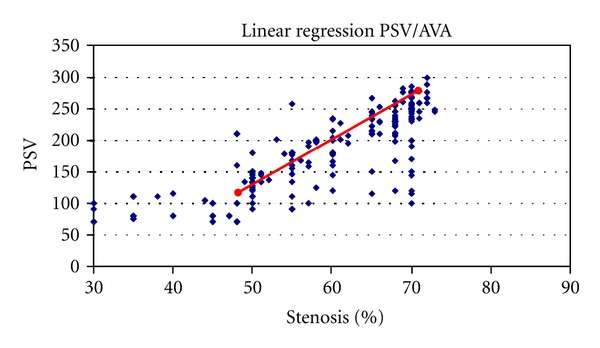
Scatter plots of absolute PSV measurements and percentage of carotid stenosis using MDCTA-AVA software measurements. The linear regression analysis showed a significant correlation between MDCTA-AVA percentage stenosis and PSV (*r* = 0.88).

Linear regression analysis showed a good linear correlation between MDCTA-AVA software percentage stenosis and PSV in grade of stenosis evaluation (*r* = 0.88) (Figure 6). Comparing carotid PSV with the grade of stenosis obtained by AVA analysis, we obtained a main PSV value corresponding to a grade of stenosis of 50% (133,6 cm/sec) and grade of vessel stenosis of 70% (268 cm/sec).

The sensitivity, specificity, positive predictive value (PPV), and negative predictive value (NPV) were 93%, 82%, 97%, and 75%, respectively.

## 4. Discussion

Several studies have shown that carotid artery degree of stenosis is a critical parameter in the evaluation of stroke risk. Many trials showed that the risk of ischemic events increases with the degree of stenosis and can be markedly reduced with endarterectomy or carotid artery stenting (CAS) [8, 10, 23]. 

Recently new parameters other than degree of stenosis have been shown to be important markers for the stratification of the risk of stroke, although the degree of stenosis is still considered the leading parameter for choosing a specific option [16, 24]. A correct, reproducible method for evaluating carotid stenosis is the mean target.

Catheter angiography is the definitive examination for determining carotid stenosis, but the risks and costs of this procedure have prompted the development of noninvasive techniques such as ultrasonography and TC [18]. Moreover DSA is a biplanar examination that could not permit obtaining the smallest diameter of the vessels with respect to the CT evaluation that allows obtaining multiplanar vessel assessment.

Currently, noninvasive tests yield excellent images of the carotid arteries, and they are completely substituting preoperative carotid digital subtraction angiography (DSA). Doppler US is by far the most common imaging examination performer worldwide to aid in the diagnosis of carotid disease. Given the prevalence of patients with carotid disease and the frequency with which patients are referred for carotid imaging, the annual number of carotid US examinations performer is considerable [25].

This imaging modality is increasingly becoming the only examination performer before surgical intervention. It was estimated by the panelists that as many as 80% of patients in the United States undergo carotid endarterectomy after a US examination as the only preoperative imaging study. In our experience more that 1004 CAS, all patients were submitted to CT or MR evaluation pre-stent [23, 26–29]. Therefore, it is of utmost importance that information provided by the US examination be reproducible and reliable [30].

We decided to compare DUS parameter (PSV) with 64-row MDCTA AVA results because although DUS is a reliable method for screening carotid artery stenosis, in our centre, preoperative patient evaluation is based on both DUS and CTA. CTA examination permits studying the level of grade of the stenosis, in addition to morphology of the aortic arch, morphology of the plaque, and the intracranial vascular anatomy. 

Grant et al. showed that the Power Doppler imaging is likely to be a reasonably accurate and cost-effective screening examination for carotid artery stenosis in asymptomatic population [31].

Similar results are demonstrated in Grogan et al.'s study [32]. Many imaging and Doppler parameters are currently used at various laboratories for the evaluation of ICA stenosis, including ICA PSV, ICA EDV end diastolic velocity, and ICA/CCA (common carotid artery) PSV ratio, CCA EDV, and ICA/CCA EDV ratio. The application of these parameters for diagnosis of ICA stenosis varies from laboratory to laboratory and sometimes within a given laboratory. The panel suggested that the ICA PSV and the presence of plaque on gray scale and/or color Doppler US images are the parameters that should be used when diagnosing and grading ICA stenosis. In this study we analyzed the PSV value about the Doppler velocity rises in direct proportion to the degree of stenosis and flow velocity. Then the degree of stenosis estimated by using ICA PSV and the degree of narrowing of the ICA lumen on gray-scale and color Doppler images should be similar as showed by Grant et al.'s study [30]. According to the basic principle of flow dynamics, Poiseuille's law, the amount of blood flow in a vessel is proportional to the fourth power of the cross-sectional diameter and to the cross-sectional area, assuming there is a constant stenosis length. Many authors reported suboptimal accuracy of US in the evaluation of stenosis degree; several critical errors can occur and the number of false-negative for stenosis 50%–70% can be high [33]. US is operative dependent and not reproducible in practice. In many settings, interpretative criteria for carotid stenosis are indiscriminately applied or the interpreter are uncertain about exactly how to make the diagnosis of carotid stenosis. About this we compared PSV values with grade of stenosis obtained by MDCTA-AVA analysis. Quantitative information can be easily obtained with new automated 3D CTA analysis software. CT angiography has evolved along with the technologic advances of CT hardware and software. Modern CT angiography, performed with multidetector high-speed CT hardware and evaluated with 3D reformatting software, accurately and reliably depicts carotid disease, and allows for direct quantification of carotid stenosis [33]. The accuracy of stenosis measurement depends on the scanning plane, which ideally should be perpendicular to the carotid artery, used to obtain magnified transverse oblique images. Some authors consider that calcified plaque could be a limitation of CT angiography. This limitation should be avoided when multiplanar volume reconstruction is used, even when circumferential calcified plaques are present. With this technique we initially visualized the whole bifurcation, including calcifications. Then decreasing the volume reconstruction, we clearly visualized the residual lumen at the maximal part of the stenosis, even if it was located near intraluminal calcifications. If multiplanar volume reconstruction is not available, transverse oblique reconstruction was used. Calcifications should not, therefore, be considered limitations of CT angiography [34]. Cross-sectional imaging obtained with modern three-dimensional imaging can evaluate complex lesion morphology, and thus the assessment of area stenosis has become feasible [3].

3D angiographic data providing a number of display, measurements, and batch filming/archive features to study user selected vessels which include but are not limited to stenosis analysis, thrombus pre-/post-stent planning procedures and directional vessel tortuosity visualization. We considered AVA analysis as a good and objective investigation that permits reproducible evaluation of stenosis determining automatically regular cross-sectional images, against in several studies the degree of stenosis was evaluated by MDCTA-NASCET measurements obtained with oblique axial images normal to lumen center line elaborated by using MPR reconestruction [3]; moreover, many centers used MDCTA MIP (maximum intensity of projection) or VR (volume rendering) recostructions that they could overnstimate the degree of stenosis. Then 64-row MDCTA AVA software is a highly accurate and precise technique for determining the percentage of stenosis from 50% to 70%.

Our study presents a small prospectical cohort of patients with stenosis ranging f stenosis ranging from 40% to 80%. This could be considered a limit of our investigation: in fact, the correlation of duplex criteria with AVA analysis in preocclusive stenosis or cases where the distal segment of the carotid is partly collapsed was not evaluated. Further studies with larger population are necessary to obtain these results.

## 5. Conclusion

The outcomes of this work suggest that Duplex ultrasound evaluation is a good assessment in high-risk stroke population screening related to the evidence of linear correlation between PSV data and grade of stenosis from 50% to 70% obtained with 64-row MDCTA AVA software. On basis of this work, we established a main PSV value whose objective corresponds to 50% and 70% vessel grade of stenosis at AVA analysis. This may be a satisfactory result, but it necessitates obtaining other new results with a larger group of study population.

64-row MDCTA AVA software is a reliable, reproducible, and objective method to accurately evaluate the degree of stenosis.

## Figures and Tables

**Table 1:** Hemodynamic parameters described by Strandness [15] for duplex assesment of internal carotid artery stenosis.

Percent stenosis			Strandness criteria	
Normal	PSV < 125 cm/sec		No spectral broadening	End-systolic bulb flow reversal
1–15	PSV < 125 cm/sec		No or minimal spectral broadening	No end-systolic bulb flow reversal
16–49	PSV < 125 cm/sec		Marked spectral broadening	
50–79	PSV > 125 cm/sec	EDV < 140 cm/sec		
80–99	PSV > 125 cm/sec	EDV > 140 cm/sec		
